# High Performance Computing PP-Distance Algorithms to Generate X-ray Spectra from 3D Models

**DOI:** 10.3390/ijms231911408

**Published:** 2022-09-27

**Authors:** César González, Simone Balocco, Jaume Bosch, Juan Miguel de Haro, Maurizio Paolini, Antonio Filgueras, Carlos Álvarez, Ramon Pons

**Affiliations:** 1 Institut de Química Avançada de Catalunya (IQAC-CSIC), 08034 Barcelona, Spain; 2 Department of Mathematics and Informatics, Universitat de Barcelona, 08007 Barcelona, Spain; 3 Barcelona Supercomputing Center (BSC), 08034 Barcelona, Spain; 4 INTEL, 20090 Assago, Italy

**Keywords:** pp-distance computation, X-ray spectra, FPGA, OpenCL

## Abstract

X-ray crystallography is a powerful method that has significantly contributed to our understanding of the biological function of proteins and other molecules. This method relies on the production of crystals that, however, are usually a bottleneck in the process. For some molecules, no crystallization has been achieved or insufficient crystals were obtained. Some other systems do not crystallize at all, such as nanoparticles which, because of their dimensions, cannot be treated by the usual crystallographic methods. To solve this, whole pair distribution function has been proposed to bridge the gap between Bragg and Debye scattering theories. To execute a fitting, the spectra of several different constructs, composed of millions of particles each, should be computed using a particle–pair or particle–particle (pp) distance algorithm. Using this computation as a test bench for current field-programmable gate array (FPGA) technology, we evaluate how the parallel computation capability of FPGAs can be exploited to reduce the computation time. We present two different solutions to the problem using two state-of-the-art FPGA technologies. In the first one, the main C program uses OmpSs (a high-level programming model developed at the Barcelona Supercomputing Center, that enables task offload to different high-performance computing devices) for task invocation, and kernels are built with OpenCL using reduced data sizes to save transmission time. The second approach uses task and data parallelism to operate on data locally and update data globally in a decoupled task. Benchmarks have been evaluated over an Intel D5005 Programmable Acceleration Card, computing a model of 2 million particles in 81.57 s – 24.5 billion atom pairs per second (bapps)– and over a ZU102 in 115.31 s. In our last test, over an up-to-date Alveo U200 board, the computation lasted for 34.68 s (57.67 bapps). In this study, we analyze the results in relation to the classic terms of speed-up and efficiency and give hints for future improvements focused on reducing the global job time.

## 1. Introduction

Knowledge of molecular structures and their spatial distribution is fundamental for biomedicine and nanotechnology. X-ray analysis, which has long been used to determine the molecular structure and spatial disposition of crystals [1], can also be employed to investigate the structure of non-crystalline or low-crystallinity samples. The crystallographic method relies on the production of crystals; however, some molecules do not crystallize or produce only small crystals. To solve this, the whole pair distribution function has been proposed to bridge the gap between the Bragg and Debye scattering theories [2]. To obtain structural and spatial information about such samples, one approach first constructs a set of possible 3D models and then generates simulated X-ray spectra by applying the Fourier transform to each model’s particle–particle (pp) distance distribution histogram. These model spectra can then be compared to the experimental X-ray spectrum in order to find a probable structure.

The main difficulty in fitting X-ray spectrum is the iterative nature of the process, which, when there are many particles, requires the computation of large numbers of pp-distances, leading to a computationally complex problem. The general pp-distance problem can be stated as: given N particles located in a three-dimensional space, compute the Euclidean distances between all of their pairs. Since there are (N^2^−N)/2 pairs of particles, the complexity of the problem is of O(N^2^), i.e., as the number of particles N increases, the duration of the computing time grows quadratically. Hence, accelerating the pp-distance calculation is crucial for this application.

The pp-distance computation is a challenging problem in many fields of science. This type of calculation is used intensively in simulation methods of statistical thermodynamics, such as Monte Carlo and molecular dynamics (MD) [3]. In both methods, thermodynamic parameters are determined by evaluating the interaction potentials between all the particles in the system. The interaction potentials (e.g., Lennard-Jones potential [4], electrostatic potential, or gravitational potential) all depend on distance. Therefore, to calculate the total energy, it is necessary to know the distance between any two particles, i.e., to numerically solve the pp-distance problem. The process will be repeated as many times as predicted motions are desired in MD or as sampled states are evaluated in Monte Carlo simulations.

To perform pp-distance calculations efficiently, high-performance computing is required. Compared to the other possible parallelization scenarios using either Central Processing Unit (CPU) or Graphics Processing Unit (GPU) implementations, the use of field-programmable gate-array (FPGA) boards provides several advantages. The motivation for using an FPGA is to have specialized hardware that can solve tasks very quickly and efficiently. One also has the flexibility to change the design when necessary. One does not need to run any kind of task code, such as those needed on a general-purpose processor.

FPGAs are highly customizable and can be programmed as desired, even for nonparallel data operations. In addition, they have economic and environmental benefits, in that, because they normally operate at lower frequencies, they consume much less energy [5,6]. In addition, in our opinion, treating this problem with OpenCL over FPGAs facilitates the programming tasks in terms of programming development time, modifications’ flexibility and ease of understanding of the code by others.

FPGAs can have a tailored hardware that, by using only the necessary precision and a dynamic scheduling, can adapt better to the problem’s characteristics. A general-purpose hardware sometimes needs to execute complex sequences of simple code in order to map the necessary operations of the application while an FPGA can have as many as necessary units that are each just as big as necessary (e.g., a square root unit).

**Figure 1:**
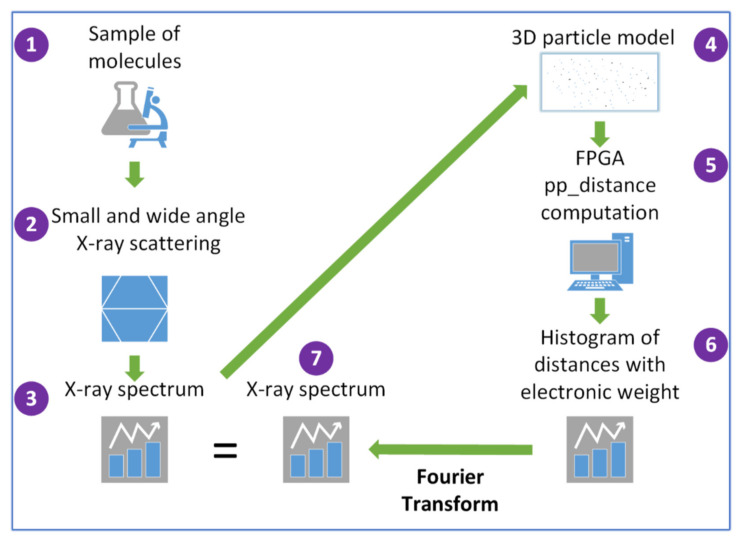
Flow chart for comparison of observed X-ray spectra with model spectra generated using field-programmable gate-array (FPGA) boards to solve the particle–particle (pp) distance problem. The numbers within the figure indicate the sequence.

The complete pipeline for the X-ray analysis of non-crystalline materials is shown in Figure 1.

The explanation of the steps relating to Figure 1:

(1) A problem sample is chosen (in principle, non-crystalline or with low crystallinity);
(2) The sample is irradiated with X-ray;
(3) The spectra are obtained after proper data treatment;
(4) A 3D model is built based on pseudo-electrons positions and contrast;
(5) All the distances between these pseudo-electrons are calculated;
(6) A histogram is built, where the X axis are the distances’ ranks, and the Y axis are the addition of their electronic weights;
(7) FT is performed on this histogram to obtain the calculated spectra; this will be compared with the experimental one and the information thus obtained is used to iterate from step 4.

FPGA capabilities are exploited in steps 5 and 6 where intensive calculations are performed.

### 1.1. Related Work

Hasitha [3] and Reuter [7] have recently attempted to compute the pp-distance for MD using FPGAs and GPUs, respectively. These authors accelerated the calculation by assuming a cut-off distance, beyond which the interaction between particles is small enough to have a negligible influence on the system’s thermodynamics. Computing X-ray spectra requires calculating the Fourier transform of the pp-distance distribution, eventually with the appropriate weighting (see Figure 1). A cut-off distance eliminates information of large distances in real space which means losing information at small q values in the inverse space and, therefore, the information concerning the shape and size of particles or domains. In addition, a reduction in the number of particles to reduce the number of pairs to be calculated is not usable because the information of short distances would be lost. Therefore, such an approximation is not suited for our application.

Instead, in this study, we minimized the time required for the pp-distance calculation using direct implementation, i.e., calculating all pairs, running on FPGA technology.

The all-pairs method in astrophysical n-body simulation, which involves the calculation of the pp-distance for gravitationally interacting systems, has recently [8] been implemented using FPGA technology. This demonstrates that current FPGA boards can provide good results for these kinds of O(N^2^) problems. In this context, Sano et al. [9] obtained the best results, for a single FPGA, on the Arria 10, with a score of 10.94 billion force calculations per second for 262,144 particles, at 180 MHz kernel frequency. A direct comparison is not possible because Sano et al. [9] not only computed the distance between all the pairs, but also this information was further used for the calculation of the total force experienced by each body.

Gu and Herbordt [10] attempted an MD implementation over an FPGA board as early as 2000, but their experiments used a Virtex-II-Pro XC2VP70 a Xilinx (USA) FPGA board, which is now considered obsolete. In our case, the experiments are performed on an Intel (USA) Arria 10 1150 DCP board [11], an Intel (USA) Stratix 10 SX FPGA [12], a Xilinx (USA) ZU102 board [13] and a Xilinx (USA) Alveo U200 [14].

To the best of our knowledge, FPGAs have not previously been used to determine the X-ray spectra. In this article, we propose a software implementation of an algorithm to achieve this, written in the C and OpenCL languages for the kernel, adapted to modern FPGA hardware. The implementation includes the design of a novel kernel optimized for interchanging data formats between CPU and FPGA.

### 1.2. Article Structure

This article presents a direct calculation of pp-distances that parallelizes the algorithm code using the unroll directive implementation over an FPGA architecture. In our case, the computation of the pp-distance entails the generation of the radial distribution function [7,15] needed to obtain the X-ray spectra via Fourier transformation. We also present an algorithm that reduces the execution time by optimizing the data transferred between CPU and FPGA, using a data-compression technique [16] that has been adapted to the specific pp-distance computation.

In Section 2, our benchmarks are explained in depth and the quantitative results are presented. These results are discussed from two perspectives: adequacy of the algorithm to the target and the speedup/efficiency of the program and kernel. Section 3 we explain our theorical 3D date model and presents the methods used to build the FPGA kernel with OpenCL [17] and the implementation of a numerical approach, written in C language using OmpSs (a high-level programming model developed at the Barcelona Supercomputing Center, that enables task offload to different high-performance computing devices) [18] to call the kernel routine.

Finally, Section 4 presents our conclusions and suggestions for future improvements.

## 2. Results and Discussion

**Figure 2:**
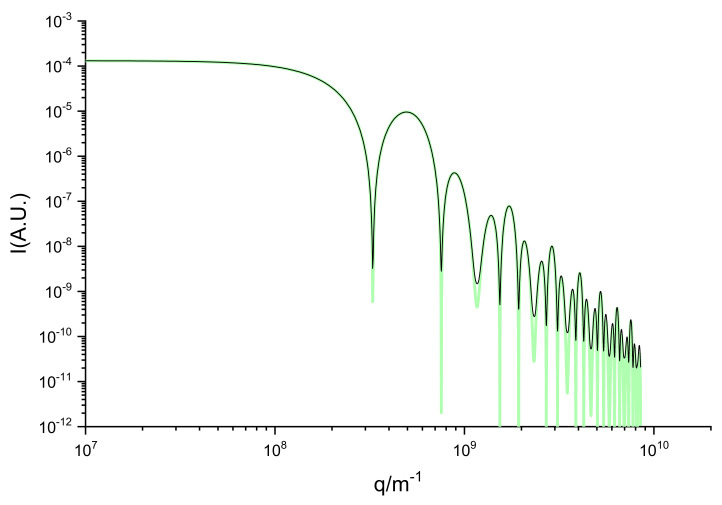
Comparison of the analytical (thick green line) and computed (black thin line) intensity spectra (as a function of scattering-vector magnitude q) of the electron distribution described in the Section 3.

The SAXS spectrum shown in Figure 2 (black lines) was determined as the sine Fourier Transform of the histogram of inter-particle distances. The histogram was obtained with a bin size corresponding to 1 picometer. Smaller bins produce noisier histograms, but the sine Fourier transform is not influenced, due to its integral nature. The decrease in bin size implies an increase in both computational cost and memory usage. The use of bigger bins results in smoother histograms and an increased computing speed, however, this comes at a cost of a smaller resolution in the large q region. To produce a good representation of the large q region, the density of points should be enough for producing a good quality histogram at small distances (few picometers). To simulate real systems, the point density should be of the order of the electron density in real matter which, for water, corresponds to 330 electrons/nm^3^ or 666 electrons/nm^3^ for glass.

The FPGA-calculated spectrum is shown in Figure 2 (black lines). The Fourier transform was computed with 4000 points; the pp-distance function was calculated in picometers. The theoretical intensity produced by the system is given analytically [19] by Equation (1):

(1) $$I\left(q\right)={\left[R_{e}{}^{3}\rho_{e}\frac{sin\;\left(qR_{e}\right)-qR_{e}cos\left(qR_{e}\right)}{{\left(qR_{e}\right)}^{3}}+R_{i}{}^{3}(\rho_{i}-\rho_{e})\frac{sin\;\left(qR_{i}\right)-qR_{e}cos\left(qR_{i}\right)}{{\left(qR_{i}\right)}^{3}}\right]}^{2}$$

where $q=\frac{4\pi }{\lambda }sin\left(\theta /2\right)$ is the magnitude of the scattering vector with λ as the wavelength of the radiation and θ as the total scattering angle (identical to 2θ in the diffraction context); *R_i_* and $\rho_{i}$ are the radius and electronic density contrast of the core; and *R_e_* and $\rho_{e}$ are the total radius and shell electron density contrast, respectively.

The green line in Figure 2 corresponds to Equation (1) with the same model parameters as the FPGA spectrum, using only a multiplying factor for the fit.

**Figure 3:**
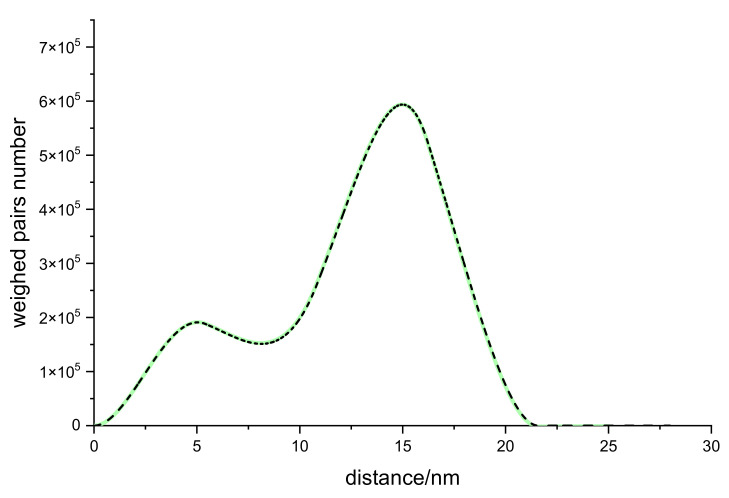
Histogram of the inter-particle distances of the system in Figure 2. Computed results (dashed black); numerical inverse Fourier transform of the analytical function (solid green).

In Figure 3, we show in dashed black the histogram from the FPGA data, and in solid green, the inverse Fourier transform of the analytically calculated spectrum with the same parameters.

The intensity function was calculated with 100,000 points up to q = 8 × 10^9^ m^−1^. It is clear from Figure 2 and Figure 3 that the FPGA results agree with the analytical results for the intensity, and that the description of the histogram is accurate at all distances in real space.

The same method can be applied to the determination of X-ray spectra of small crystals as we have shown in a recent conference paper [20] where we compare the experimental spectra of silver behenate with the calculated spectra using pseudo-electrons for a 10 × 12 × 17 nm^3^ nanocrystal.

### 2.1. Experimental Setup

We ran the OpenCL algorithm for exhaustive tests on the FPGA board, an Intel Arria 10 1150 DCP board [11] on an Intel Xeon CPU E5-2690 v4 @ 2.60GHz computer with 56 processors and 132 Gigabytes of memory. The optimized kernel was tested on two FPGA platforms—the Intel Programmable Acceleration Card with Intel Arria 10 GX FPGA and Intel FPGA Programmable Acceleration Card D5005, featuring an Intel Stratix 10 SX FPGA [12]. The kernel for the FPGA was compiled with the Intel FPGA SDK for OpenCL version 20.1 using as backend the Intel Quartus compiler [21] version 17.1.1 for the first card and version 18.1.2 for the second.

The OmpSs@FPGA environment with the Picos Hardware Runtime has been tested on a Xilinx Zynq UltraScale+ MPSoC ZCU102 [13]. The system on chip is composed of four ARM Cortex-A53 cores that run at 1.1 GHz, a Xilinx ZU9EG FPGA and a main DDR4 memory of 4 GB. The board is booted using the Ubuntu Linux 16.04 operating system. The tools used to generate the application bitstreams and binaries are: Vivado Design Suite 2018.3, GNU C/C++ Compiler 6.2.0, PetaLinux Tools 2019.1 and OmpSs@FPGA tools from release 2.2.0 with the Picos Daviu version. Table 1 shows the main characteristics of the different systems.

### 2.2. Standard Runtime Library

Specific Results of the Benchmarks

The tests were completed, over the Arria 10 DCP, for the following parallelization UF:

- UF = 1 (no parallelization)
- UF = 8, 16 and 32 degrees of parallelization

**Figure 4:**
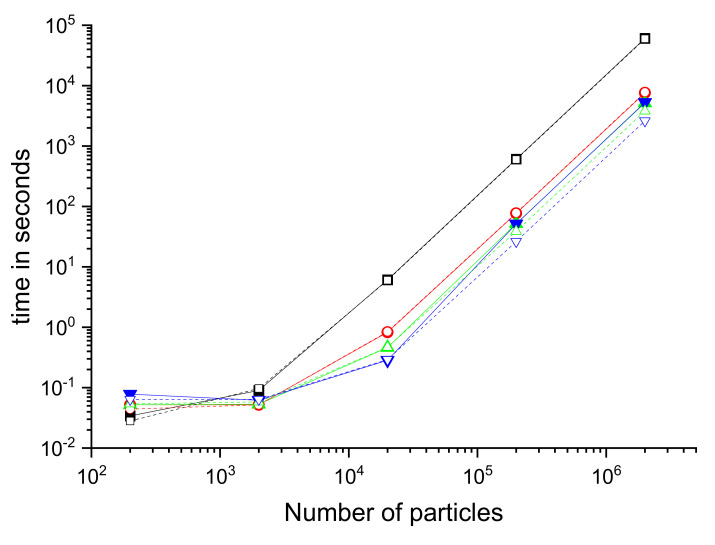
Comparison of execution times for kernels with different unroll factors (UF = 1 black squares, UF = 8 red Circles, UF = 16 green up triangles, UF = 32 blue down triangles) and different transmission format (full lines and symbols float 32 bit and dashed lines and empty symbols unsigned short 16 bit).

Results for both the 32 and 16-bit transmission formats are shown together in Table 2 and in Figure 4.

The frequency of the kernels is around 100 MHz. This fact is important because the low frequency implies that the energy consumption of FPGAs is low in comparison with other electronic devices such as CPUs. These results could be improved further by increasing the frequency slightly by using different seeds at kernel compilation.

### 2.3. Relevance of Speed-Up and Efficiency

**Figure 5:**
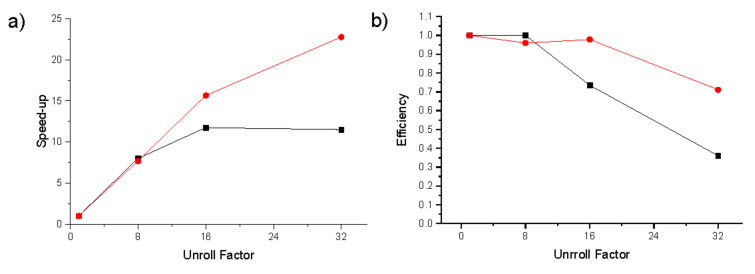
(**a**) Speed-up as a function of unroll factor for kernels with 16-bit (red circles) and 32-bit (black squares) transmission. (**b**) Efficiency as a function of unroll factor for kernels with 16-bit (red circles) and 32-bit (black squares) transmission.

The speed-up corresponds to the ratio between run-time for the execution of the non-parallelized code and that of the parallelized versions. We can build the speed-up graph by combining the runtime results using the two kernels with different data format on one graph, as shown in Figure 5a.

As could be expected, the more parallelization we used, we needed a higher amount of simultaneous data. This high amount of data can saturate the FPGA transmission data resources to reach the limits of the FPGA hardware. With the 32-bit transmission code we can see, in Figure 5a, how the amount of data demanded represents a bottle neck:

- With the UF = 16, the speed-up does not maintain the linear scaling, decreasing from the optimal expected 16 to 11.75;
- With the UF = 32, we obtain an even worse performance than with the UF = 16. The speed-up falls to 11.50, which is far away from the expected optimum of 32. That the same algorithm with less data transmission has a better speed-up rate indicates that the data transmission caused the application to collapse.

The efficiency corresponds to the ratio of the speed-up over the parallelization factor. The efficiency issues reported here, especially for the 32-bit transmission kernel (Figure 5b), are probably caused by the memory access patterns of the four read units in the kernel, which can force stalls and limit the efficiency of the memory interface. This and other tuning issues are discussed in Supplementary Materials.

Independently of the FPGA characteristics, we can deduce that an improvement in data transmission implies the possibility of data parallelism and increases speed.

**Figure 6:**
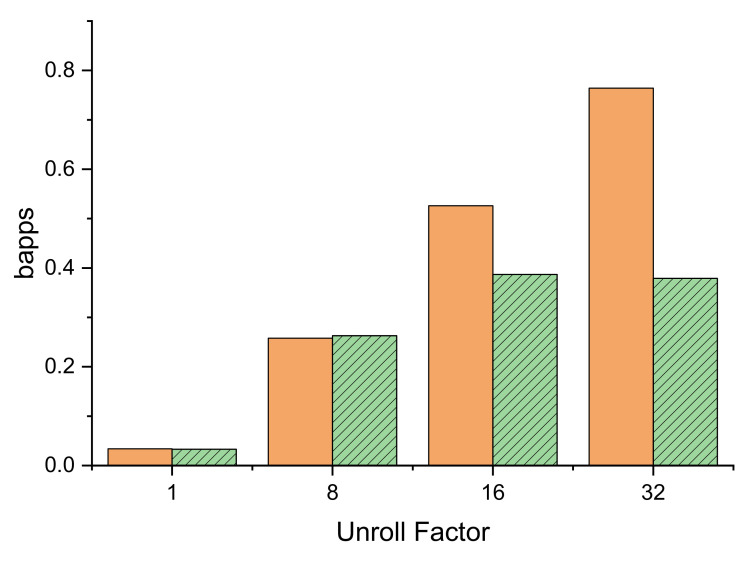
Processing rates per billion atom pairs for 16-bit (plain orange) and 32-bit (dashed green) transmission formats at unroll factors from 1 to 32.

To compare performances, we can express the speed of our computation in units of billion atom pairs per second (bapps) [7]. The ratio of the performances of the kernels, taken over many particles and expressed in bapps, could help us to extrapolate time results and predict how long an algorithm we will run with a larger (or smaller) number of particles. Figure 6 shows such a comparison for both transmission formats and various UFs.

### 2.4. Improved Picos Runtime Library

This section presents the results obtained on the Xilinx Z102 board using the OmpSs@FPGA infrastructure. This approach is characterized by two levels of parallelization. The first one, as in the previous runtime, is the UF of each Distance kernel accelerator (see Algorithm 1 in Supplementary Materials). This UF states how many particle-to-particle comparisons are performed in parallel inside the hardware accelerator. In addition, another extra level of parallelization is executed between different hardware kernels at the task level. The OmpSs runtime automatically parallelizes the tasks among the available kernels if both the degree of parallelism in the algorithm and the available hardware resources are adequate. In the present application, these conditions are met for all the Distance kernels available and up to as many Aggregate kernels. However, as seen in Figure S5, the Aggregate tasks are faster than the Distance tasks. We have therefore chosen to add as many Distance kernels as possible to fit in the FPGA while maintaining only one Aggregate kernel that executes all of the Aggregate tasks sequentially. In our case, we have three parameters: UF, kernel instances and frequency. So various combinations are tested. In general, we tend to have a tradeoff between UF, kernel instances and frequency of operation. The best combination will depend on the accelerator and the device.

#### 2.4.1. Determining Optimum Frequency and FPGA Usage

In the OmpSs@FPGA environment, the first task is to determine how many kernels can fit in the FPGA and at what frequency they should work.

Although it is possible to choose different frequencies individually for each kernel (up to a certain point, depending on the capabilities of the FPGA), we have chosen to make the whole system work at the same speed for the sake of simplicity. In the current design, most resources are devoted to the Distance kernel and consequently the other hardware resources do not obtain any significant advantage by working at a different frequency.

The second task is to determine how many resources should be used in each of the kernels. This task is influenced by the previous one, as the same kernel working at two different frequencies could use different amounts of resources. In Table 3, the different configurations tested in the FPGA are summarized. All the configurations shown are the largest configurations that can be fitted in the FPGA by the vendor’s place and route tools. For each configuration, the Maximum working frequency is listed. The number of Distance kernels in the configuration, their unroll factor and the FPGA resources used by the configuration are also detailed. The resources used are Flipflops (FFs), Look-Up-Tables (LUTs), Multiply Accumulator units (MACCs) and Block RAMs of 36Kb each (M36Kb). To find each configuration, a number of Distance kernels (given in the Units column of Table 3) have been selected for each frequency. Then, the UF of each of these Distance kernels has been augmented as much as possible while still fitting in the FPGA. The column “FPGA resources used” lists those resources used in the whole design, including and in addition to the Distance kernels, one Aggregate kernel, one FPGA_Main kernel and one POM hardware manager. The percentage of the resources used is included in parentheses.

**Figure 7:**
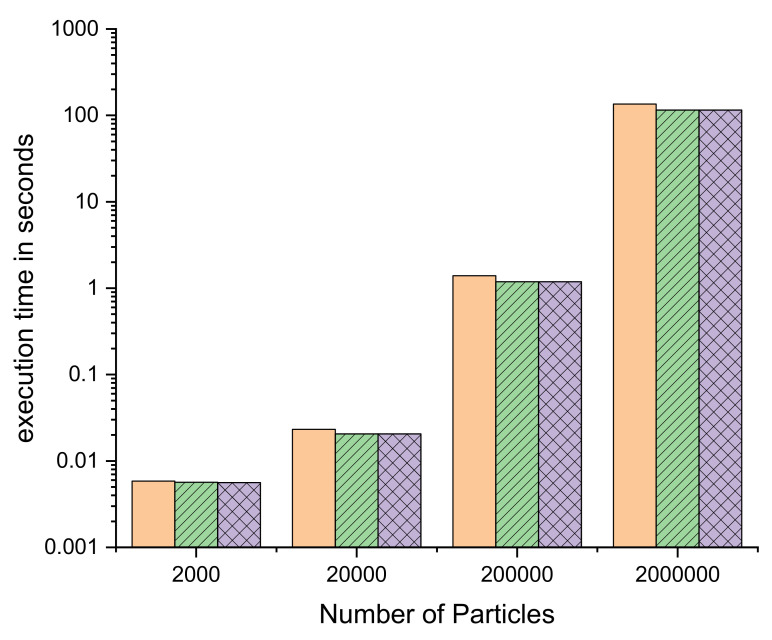
Execution time as a function of number of particles at different frequencies: 4 × 36_214 MHz. orange full column, 4 × 36_250 MHz. green dashed column and 4 × 30_300 MHz. lilac crossed column.

The data in Table 3 show that lowering the working frequency below 250 MHz does not save enough resources to permit the inclusion of more computing units (either by adding more Distance Kernels or by increasing their UF). This can be seen by comparing row 4 × 36_214, row 4 × 36_250 and row 4 × 32_300. Although changing the frequency from 300 to 250 MHz produces an increase of four in the UF for each kernel, further lowering it to 214 MHz does not produce any additional gain, as there are no more available resources in the FPGA (a 99.8% occupancy in the M36Kb blocks means that there are only 2 blocks not used in the design, out of a total of 912). Figure 7 shows the time comparison of these three designs (for four units of Distance kernels, which achieve the best performances) for various numbers of particles, on a logarithmic scale where the performance loss at 214 MHz can be appreciated. On the other hand, the tools have failed to produce a competitive working design at frequencies higher than 300 MHz.

In Figure 7, we can observe the effect of the number of particles on the execution time for the three different frequencies used and similar unroll factors. Note that the number of operations scale with the square of the number of particles. It is apparent that a small number of particles results in lower productivity than bigger ones due to a number of fixed operations that do not depend on the total number of particles. Otherwise, the difference between different frequencies is small but not negligible because the scale is logarithmic. We also have to consider that kernels are compute-bound, therefore its speed is directly proportional to the number of computation units times UF times frequency. That is, at 300 MHz, performance is proportional to 4 × 30 × 300 while that of 250 MHz is proportional to 4 × 36 × 250 (i.e., practically the same). The drop in frequency is partially compensated by the increase in accelerator UF.

#### 2.4.2. Performance Results

**Figure 8:**
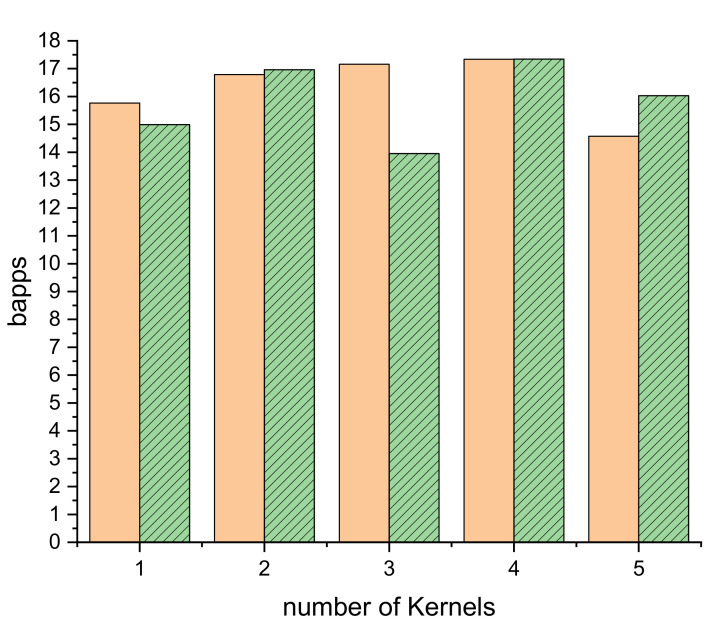
Bapps for OmpSs@FPGA designs with different number of Distance kernels for 2 million particles. Full orange column running at 250 MHz and dashed green column running at 300 MHz.

Figure 8 shows the performance in bapps for the 250 and 300 MHz designs shown in Table 3. It can be seen that both the designs obtain good performances. The smaller clock frequency seems to have more performance stability, but the higher clock frequency is able to deliver better performances in the most optimum designs. The optimum design point changes between designs with the number of Distance kernel accelerators, but both of them give the best result at three or four kernels (with a UF of 44 at 300 MHz or 36 at 250 MHz, respectively). The difference between three and four kernels is slight at both frequencies and depends on where the vendor’s place and route tools find the optimum point. In both cases, there is significant loss of performance when using only one large kernel or more than four. A single kernel suffers due to memory contention, as the kernel is either accessing the memory or computing. Two or more kernels can alternate memory access (by one kernel) and computation (in the remaining kernel(s)); this is shown in Figure S6, where the blue bars overlap in time (vertical) with yellow and red zones. On the other hand, when there are too many kernels, the extra resources necessary to implement each extra accelerator are subtracted from the set of resources available for computation.

One of the limitations of the OmpSs@FPGA environment is that being an academic effort, it is only available for a limited set of FPGA boards. To make a proof-of-concept of the potential of the Picos design in larger boards, a limited test has been implemented in an Alveo U200 card (that features 6840 MACCs) [14]. The same design tested in the ZU102 board but with 12 Distance kernels (unroll 40) and maintaining 1 Aggregate kernel working at 250 MHz achieves 34.68 s for 2,000,000 particles, resulting in 57.67 bapps while using 94.65% of the FPGA MACCs. As the porting of the OmpSs@FPGA framework to the Alveo boards is still an ongoing work, this number is expected to grow as a more mature implementation that allows a more thorough testing is developed. This value of 57.67 bapps can be compared with 11 bapps obtained by Sano et al. [9] for force calculations with using cut-off distances (although we are talking about different technologies, Alveo U200 vs. Arria 10 with more than 5 years difference). A more direct comparison can be made on the histogram construction by A. Leonardi and D.L. Bish [22] which achieved 0.55 bapps using GPU technology or up to 30 bapps using a cluster with 120 Tesla K20.

More information regarding an optimized OpenCL implementation can be found in the Supplementary Materials where a detailed description of OmpSs is made, including the parameters (Figure S1), compilation process (Figure S2), task dependence (Figure S3), application flow (Figure S4), trace execution (Figures S5 and S6), implementation details of the configurations tested (Table S1), execution time for an optimized kernel (Figure S7, and Table S2) speed-up and performance (Figures S8 and S9) [12,23,24,25,26,27,28,29].

## 3. Methods and Materials

### 3.1. Model: Data

**Figure 9:**
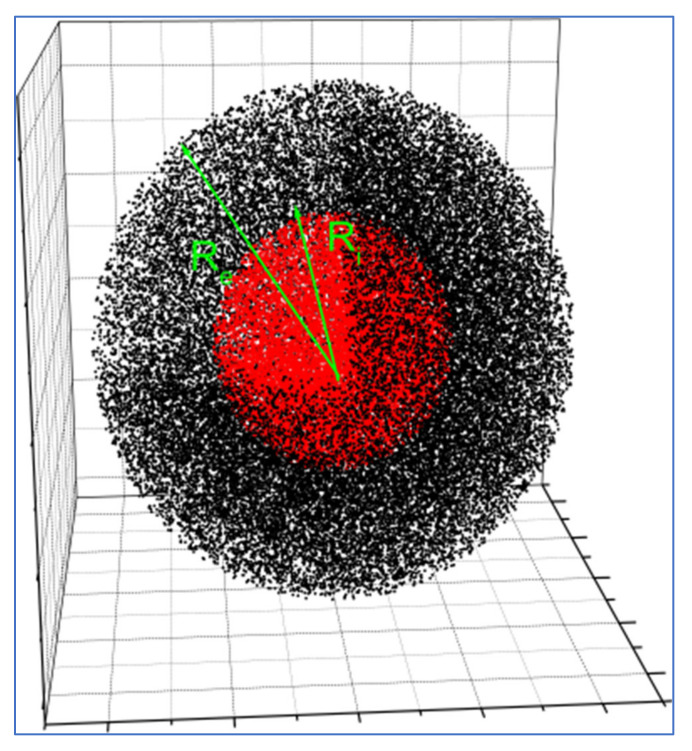
Distribution of sample of particles. R_i_ and R_e_ are the radius of the core and the total radius of the sphere, respectively. Colors of the dots represent the negative (red core) or positive (black shell) contrast corresponding to each pseudo-electron.

As a test for our approach, we chose a large random distribution of 2 million pseudo-electrons forming two concentric spheres in a core-shell disposition (Figure 9). Because of its very high symmetry, this model has an analytical solution and, therefore, it is straightforward to determine the reliability of the method. Our computational goal was to compute the pp-distances of all distinct pairs. The pseudo-electrons were randomly distributed in a sphere containing an inner core surrounded by a shell with a different electron density, representing a simple nanoparticle. The number density of pseudo-electrons was the same for the core and the shell. The core radius R_i_ was 5.4 nm, and the total radius Re of the sphere was 10.8 nm. The core had negative contrast, i.e., it had a low electron number density relative to the surrounding water: $\rho_{i}=-0.08\;electrons/{Å}^{3}.$ The shell had positive contrast: $\rho_{e}=0.07$. Algorithm 2 (see Supplementary Materials) was used to generate a source dataset of two million random positions compatible with these densities. The model particle sizes fell within the usual sizes encountered in nanoscience and were suited for Small Angle X-Ray Scattering.

### 3.2. Materials: Program, Kernel, and Data Formats

A direct implementation of the pp-distance problem has always been a challenge [30]. Algorithm 3 (which can be found as Supplementary Materials) shows the pseudocode of a program that computes all the distances between N particles.

In our approach, the pp-distance computation consisted of two parts:

(1) A main program—*spectra.c*—which read and pre-processed the data;
(2) A kernel routine implementing Algorithm 1, which was called the main program.

Note that the pp-distance computation implemented is the main iteration of the whole X-ray matching computation (see Figure 1) and is very time consuming. Therefore, the full application time is defined by the time of this computation.

It is also important to note that both the program and kernel need to use the same data format (16 or 32 bit). As the execution time of the kernel in the FPGA is affected by the length of the format of the transmitted data (the shorter the data format, the faster the execution), we designed two implementations for interchanging data formats between the CPU and FPGA. The first one used a standard 32-bit format, while the other was based on a tailored 16-bit unsigned short format.

Considering that the largest possible inter-particle distance in our model was 21,600 picometers, we did not need to accommodate coordinates larger than such a value. Therefore, we were able to convert the 32-bit float data to 16-bit unsigned word data in two steps:

(1) We transformed the set of floats to integer values by changing the float values from meters to picometers (i.e., multiplying our data by a factor of 10^12^), because the source data were in meters and the resolution of the histogram was in picometers. The resolution of the histogram must be congruent with the precision of the computed distances. That is, a histogram with 10^−12^ resolution needs distances calculated with the same precision;
(2) We made positive all of the 3D coordinates of the particle-model by adding the maximum possible value of the negative coordinate to all the values. The fundamental structure of the model was unaffected by these changes.

Hence, we were able to transform every model datum into an unsigned short, which can represent any integer from 0 to 65,535. This approach can only be applied to evaluate models with maximum inter-electron distances smaller than 65,536 pm. Clearly, our model involved no distances exceeding or even approaching this threshold.

### 3.3. Techniques: Kernel Structure

In the first approach, the kernel was implemented in the OpenCL language, which permitted us to write C-like code for FPGAs in much the same way that C-CUDA permits C-like code for GPUs. We used unroll factor (UF) (a feature of the OpenCL that allows the OpenCL compiler to replicate in parallel a loop body multiple times into the FPGA hardware) in our algorithm to introduce different degrees of parallelism. An unroll factor of 1 means that no parallelization is used, an unroll factor of 2 means that the loop is divided in two (i.e., a loop running from 1 to N is divided in a loop running from 1 to N/2 and a loop running from N/2 + 1 to N and the hardware duplicated, and these two loops are executed in parallel) reducing the execution time of the loop almost to half of the non-parallelized code (unroll factor 1). The programmer can increase the unroll factor, provided the FPGA has enough hardware resources to increase the parallelization.

**Figure 10:**
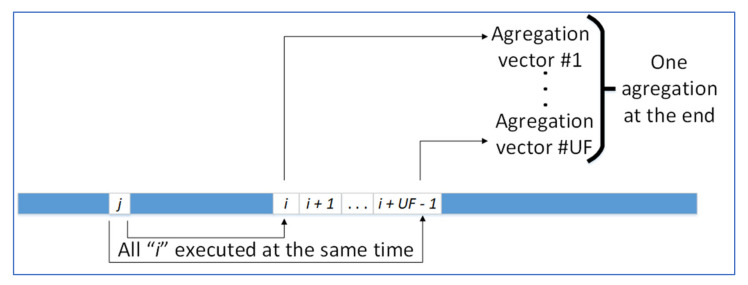
Schema of how the aggregation works with different unroll factors (UF). Here, *j* is a test particle, *i* another particle. Each time, UF operations are executed in parallel.

The program variable UF controlled the unroll factor parameter, which will take the values 1, 8, 16 or 32, as we explain later. Thus, we needed to have exactly UF aggregation vectors, one for each parallel thread. These aggregation vectors gave us the ability to add in parallel the different electronic weights without any overlap of memory access (see Figure 10), because the 1, 8, 16 or 32 hardware units forming the FPGA kernel updated the information in these aggregation vectors simultaneously.

In Figure 10, all distances from particle *j* have to be calculated. Instead of calculating the distance to every other particle sequentially (to particle *i*, to particle *i+1*, to particle *i+2* …), we used loop unrolling and calculated the distances to a set of UF particles from *i* to *i + UF−1*, storing the results in the UF aggregation vectors. The value of *i* is then increased by UF units until as many remaining particles have been processed by this method as possible.

Whenever we ran the unroll, we usually found several remainder cases (which appear when the number of cases is not an integer multiple of UF factor) that we had to compute individually at the end. We considered this technique as a “dynamic unroll”, because the number of remainder cases when we applied the unroll was not always the same for every particle and so had to be determined by the program.

At the end of the kernel there was an aggregation from the UF vectors to the returned value vector, as shown in Algorithm 3 in Supplementary Materials. The UF parameter affected the algorithms as follows:

- When no parallelization factor was used (UF = 1), we had a sequential algorithm;
- The larger the parallelization value of the UF used, the more memory from the FPGA (for the aggregation vectors) was consumed, and the larger the demand for parallel data. These two factors, memory and data transferred, impacted on the capacity of parallelization within the FPGA and limited the threshold of the UF parameter.

In order to obtain a better performance from the algorithm, we implemented a new approach using the OmpSs@FPGA version of OmpSs. This approach divided the kernel computation into different tasks that were executed in a dynamically computed order on different accelerators. More information regarding the OmpSs tool and a second kernel organization can be found in the Supplementary Materials (Figures S5 and S6).

## 4. Conclusions

To bridge the gap between Bragg (i.e., classical crystallography) and Debye scattering theories to resolve the structure of biological molecules and other nanostructures, the whole pair distribution function has been proposed [2]. In this work, we propose a solution based on the computation of pseudo-electron histograms, which we have shown can be computed effectively by using FPGA to highly accelerate the calculation. This procedure and technology allow for fast calculation of X-ray scattering spectra generated from 3D models, making this calculation usable in iterative fitting procedures. A single spectra calculation could be accelerated to last for 35 s for a 2 M particles’ model.

The best efficiency/cost ratio in our experiments has been obtained with Xilinx FPGAs as they are cheaper than the Intel Altera FPGAs used and obtain better performance results. However, the programming model used has been different for each vendor technology (OpenCL for Intel, OmpSs for Xilinx) due to the tools’ limitations, not due to any intrinsic hardware limitation. Without more experiments that are out of the scope of this paper—as we are not designing new FPGA tools—it is difficult to know if the advantages obtained are due to the hardware or to the capabilities of the toolchain. It must be clear that we are not implying that Xilinx is better than Intel, in fact we think that the different results are due to the use of OmpSs vs. OpenCL. Based on our experience, we can argue that if you want a better performance, the best is Intel + AlveoU200, but if you want a lower price, the best would be ZU102. Using OmpSs@FPGA [25,27] would be appropriate in the case of Xilinx and OpenCL in the case of Intel.

By using different parallelisms on the FPGA, we found that the data transmission speed between the FPGA and CPU limits the overall speed when using higher parallelism. The use of a data compaction procedure allowed the parallelism to be pushed further without unacceptable loss of precision. Therefore, as less data are transmitted between the CPU and FPGA, better results are obtained.

On the other hand, focusing on local data and managing tasks locally seems to be a good way to conserve computational resources on the FPGA. By creating a small set of tasks, the balance between task and data parallelism can be easily changed to one that reaches the optimum of the fabric.

The performance of the OmpSs@FPGA approach efficiently leverages FPGA resources and consequently depends on the size of the FPGA used. Future work will address extending the current implementation to newer, bigger FPGA fabrics. With this new hardware, we will have the opportunity to increase the internal kernel parallelism, but also to implement other, different approaches. One of the challenges could be finding an easier method to reduce or compress the data traffic that the CPU and FPGA share, because that will allow the use of more unrolled parallelism. In addition, joining FPGAs with other devices to run the application could improve the performance per particle immediately through the balanced distribution of work between these different devices. One of the limitations of the OmpSs@FPGA environment is that being an academic effort, it is only available for a limited set of FPGA boards.

## Figures and Tables

**Table 1:** Experimental hardware characteristics.

	CPU		FPGA			
System	Name	Freq	Name	System Logic Elements ^¥^/Cells ^§^ (k)	FPGA Memory (Mbits)	Multipliers (18 × 19 ^¥^/18 × 27 ^§^)
Arria 10 DCP	Intel E5-2690	2.6 GHz	Arria 10 1150	1506 ^¥^	65.7	3036 ^¥^
D5005 PAC	Intel Bronze 3204	1.9 GHz	Stratix 10 2800	2800 ^¥^	244	11,520 ^¥^
ZU102	ARM A53	1.1 GHz	ZU9EG	600 ^§^	38	2520 ^§^

The symbols ¥, § mark compatible units.

**Table 2:** Kernel time in seconds for the different executions. UF: unroll factor.

Number	UF Value							
of	32-Bit Format				16-Bit Format			
Particles	1	8	16	32	1	8	16	32
200	0.034	0.052	0.052	0.078	0.028	0.044	0.054	0.064
2000	0.090	0.052	0.053	0.062	0.097	0.052	0.058	0.064
20,000	6.112	0.825	0.463	0.284	6006	0.845	0.467	0.292
200,000	607.787	76.625	51.967	51.297	595.847	78.12	38.816	26.35
2,000,000	60,749.326	7600.464	5171.473	5278.310	59,547.493	7756.195	3805.655	2616.741

**Table 3:** Characteristics of different configurations tested in the Xilinx FPGA.

Configuration		Distance Kernel		FPGA Resources Used (%)			
Name	Frequency	Units	Unroll	FFs	LUTs	MACCs	M36Kb
1 × 136_300	300	1	136	260,002 (47.4%)	205,172 (74.9%)	1861 (73.8%)	789 (86.5%)
2 × 60_300	300	2	60	279,974 (51.1%)	183,675 (67.0%)	1841 (73.1%)	733 (80.4%)
3 × 44_300	300	3	44	261,046 (47.6%)	178,209 (65.0%)	1776 (70.5%)	800 (87.7%)
4 × 32_300	300	4	32	261,068 (47.6%)	164,778 (60.1%)	1615 (64.1%)	813 (89.1%)
5 × 22_300	300	5	22	287,731 (52.5%)	193,703 (70.7%)	1748 (69.4%)	773 (84.8%)
1 × 144_250	250	1	144	283,774 (51.8%)	234,627 (85.6%)	1969 (78.1%)	686 (75.2%)
2 × 72_250	250	2	72	281,892 (51.4%)	219,734 (81.2%)	1991 (79.0%)	854 (93.6%)
3 × 48_250	250	3	48	289,177 (52.7%)	223,735 (81.6%)	2013 (79.9%)	878 (96.3%)
4 × 36_250	250	4	36	297,852 (54.3%)	237,057 (86.5%)	2035 (80.7%)	910 (99.8%)
5 × 24_250	250	5	25	269,588 (49.2%)	201,927 (73.7%)	1733 (68.8%)	824 (90.3%)
4 × 36_214	214	4	36	297,680 (54.3%)	237,127 (86.5%)	2035 (80.7%)	910 (99.8%)

## Data Availability

The data presented in this study are available in the article and Supplementary Materials.

## Supplementary Materials

Supplementary Materials can be downloaded at: https://www.mdpi.com/article/10.3390/ijms231911408/s1.

## Abbreviations

3D	Three-dimensional.
bapps	Billion atoms pairs per second.
CPU	Central Processing Unit.
FPGA	Field-programmable gate array.
GB	GygaByte.
GHz	Gigahertz, frequency equal to one billion hertz.
GNU	General Public License.
GPU	Graphics Processing Unit.
MD	Molecular dynamics.
MHz	Megahertz, frequency equal to one million hertz.
OmpSs	StarSs* programming model flavor targeting CPUs, GPUs and FPGAs.
pp	Particle–particle.
UF	unroll factor.
* StarSs	Task-based programming models where developers define some parts of the application as tasks, indicating the direction of the data required by those tasks.
