# Supplementary material for: High Performance Computing PP-Distance Algorithms to Generate X-ray Spectra from 3D Models
Source: Int J Mol Sci. 2022 Sep 27;23(19):11408. doi: 10.3390/ijms231911408 (PMC9570381; doi:10.3390/ijms231911408)
Supplement: Supplementary file 1 [file ijms-23-11408-s001.zip › ijms-1873247-supplementary.pdf]

International Journal of *Molecular Sciences*

Supplementary Materials

High Performance Computing PP-Distance Algorithms to Generate X-ray Spectra from 3D Models

César González [1,*], Simone Balocco [2], Jaume Bosch [3], Juan Miguel de Haro [3], Maurizio Paolini [4], Antonio Filgueras [3], Carlos Álvarez [3], Ramon Pons [1,*]

[1] Institut de Química Avançada de Catalunya (CSIC), 08034 Barcelona, Spain
[2] Department of Mathematics and Informatics, Universitat de Barcelona, 08007 Barcelona, Spain
[3] Barcelona Supercomputing Center (BSC), 08034 Barcelona, Spain
[4] INTEL, 20090 Assago, Italy
* Correspondence: cesar.gonzalez@iqac.csic.es (C.G.); ramon.pons@iqac.csic.es (R.P.); Tel.: +34-934006100 (ext. 437838) (C.G.); +34-934006150 (R.P.)

Citation: González, C.; Balocco, S.; Bosch, J.; de Haro, J.M.; Paolini, M.; Filgueras, A.; Álvarez, C.; Pons, R. High Performance Computing PP-Distance Algorithms to Generate X-ray Spectra from 3D Models. *Int. J. Mol. Sci.* **2022**, *23*, 11408. https://doi.org/10.3390/ijms231911408

Academic Editor: Alberto Pais

Received: 2 August 2022
Accepted: 22 September 2022
Published: 27 September 2022

Publisher's Note: MDPI stays neutral with regard to jurisdictional claims in published maps and institutional affiliations.

Copyright: © 2022 by the authors. Licensee MDPI, Basel, Switzerland. This article is an open access article distributed under the terms and conditions of the Creative Commons Attribution (CC BY) license (https://creativecommons.org/licenses/by/4.0/).

1.1. OmpSs

OmpSs is an academic task-based parallel programming model that serves as a forerunner of the OpenMP industrial standard. Among the main features of OmpSs already incorporated in OpenMP are OpenMP 5.0 tasking capabilities. A source-to-source compiler (Mercurium [23]) and runtime library (Nanos++ [24]), both developed at the Barcelona Supercomputing Center (BCS), are used to implement OmpSs features.

We use the programming model OmpSs developed and maintained at the BCS to integrate the kernel call in the main program. Algorithm 3 shows the OmpSs target directive ❶ and the kernel declaration ❷ in the main program, which are necessary for our kernel invocation. Please see supplementary materials, document 2, to follow at the appendix the pseudo-code ❶ and ❷ referenced in this document.

The variable N is the number of particles to be considered and F is the number of distribution positions.

Note that only the aggregation vector for frequencies h_A is returned to the CPU, because the coordinate vectors h_B1, h_B2, h_B3 and the electronic weight vector h_B4 are only read data, as shown in Figure S1.

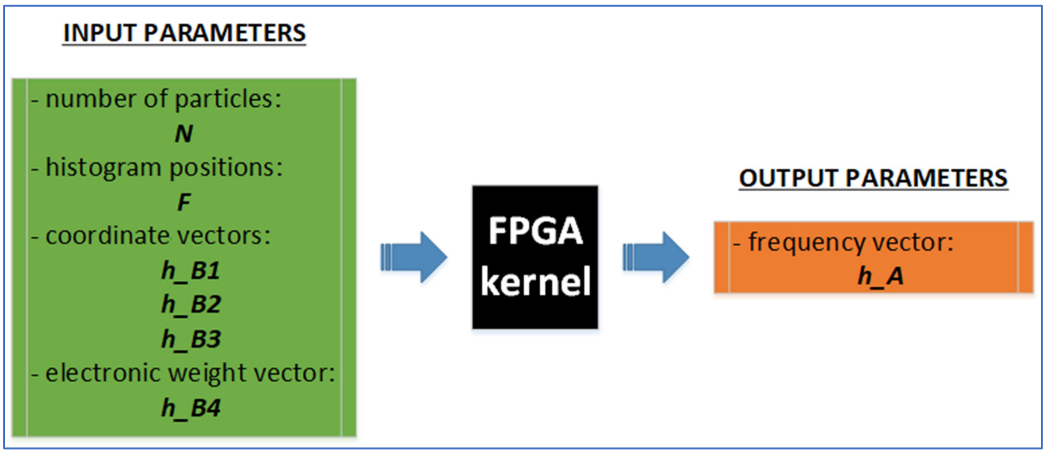

Figure S1. Input / Output kernel routine parameters.

1.2. Improved OmpSs@FPGA Framework

The second implementation relies on the OmpSs@FPGA [25] environment. The OmpSs@FPGA ecosystem evolved from the OmpSs programming model but incorporates FPGA support. OmpSs@FPGA adds support for generating tasks with the FPGA target to the Mercurium compiler. In addition, OmpSs@FPGA Nanos++ runtime handles data movement between the host and FPGA memory and offloads the tasks to the device. The ecosystem includes other tools, such as autoAIT, which combines all FPGA files generated by Mercurium and calls specific vendor tools that generate the bitstream, and the xTasks library, which provides a common application programming interface to Nanos++ for data and task management regardless of the communication protocol between the host and the FPGA. The structure of all those tools is shown in Figure S2.

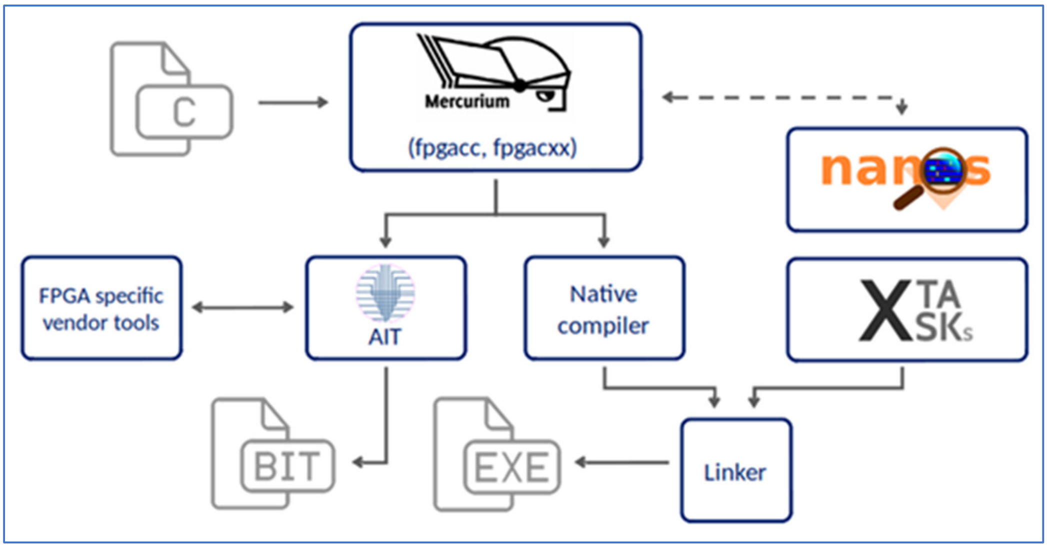

Figure S2. OmpSs@FPGA compilation process.

Algorithm 5 shows how an FPGA task is defined in the OmpSs programming model. In contrast to OpenMP, OmpSs supports the annotation of function declarations that result in a task being spawned every time the function is called. In Algorithm 5, the function Distance is annotated with the task and target directives. The device(fpga) clause means that the following task will be executed in an FPGA device instead of the host processor. The num_instances(NUMACC) clause defines the number of FPGA accelerators to be generated in the FPGA bitstream which will be available to execute tasks concurrently at runtime. The inout([BSIZE]histogram) clause defines both the dependencies that the task has and the copies (due to the clause copy_deps) that the runtime must make between the host memory and the FPGA memory before and after task execution.

The Mercurium compiler isolates the source code of each FPGA task in the application in a separate source file and wraps it in a communication wrapper. This wrapper, which has a common external interface independent of task arguments, provides instrumentation capabilities and data caching optimization. Next, the files that have been generated are provided to autoAIT in order to produce a bitstream with all the accelerators included. The communication between the runtime and the accelerator is managed by an IP instantiated by autoAIT in the FPGA bitstream to coordinate the execution of application tasks in the FPGA.

As can be seen in Algorithm 5, the code implements the histogram computation by creating a Distance task between every two pairs of blocks of points. Each task then computes the distance between all the points in its two parameter blocks. As the algorithm computes a histogram of the distance between every two particles, several histograms should be used in parallel (and then aggregated) to allow the computation of multiple

elements in parallel. In addition, some code (not shown in Algorithm 5) is needed to take care of the corner case where the two blocks provided to the task Distance are the same. The code in Algorithm 5 can be tuned by increasing the number of Distance IP kernels working in the FPGA at the same time (NUMACC) and/or by increasing the number of particles that each kernel computes in parallel (UNROLL). Also, the size of each particle block (SIZE_NB) can be adjusted to change the ratio between the computation and data communication efforts in the task.

A potential source of inefficiency in the algorithm is the large amount of FPGA area that double precision operations use in the selected board. If fewer double precision operations are used to implement the algorithm, more resources can be devoted to the other computations, thus, improving performance. One way to achieve this would be to aggregate the histograms into fixed-point histograms, provided the size of the block (SIZE_NB) is also limited to prevent overflows even in the worst possible case (when all the particle pairs in a given pair of blocks have exactly the same distance between them). However, this improvement leads to another problem: an extra kernel is needed in order to aggregate these histograms of a subset of particles into the double precision histogram that is needed as an output of the algorithm.

Algorithm 6 shows this new approximation. A new, smaller (and thus faster) task appears in order to aggregate the histogram subsets into the final histogram. This smaller Aggregate task must be executed after each Distance task and, as it is faster (only needing to add F histogram values to the total), it can be implemented with a single kernel that serves all the NUMACC Distance ones. The practical problem with this approach is that there are many fine-grain synchronizations between Distance tasks and Aggregate tasks. Furthermore, the size of each Distance task is now limited to prevent overflows in the program, leading to more numerous but smaller tasks. Figure S3 shows the dependence pattern between these tasks. Note that, as we cannot create an arbitrary number of histograms for every Distance task, there is another dependence (not shown) between each use of the same subhist vector. However, as we know the number of maximum parallel Distance tasks executed (equal to the number of Distance kernels implemented in the FPGA), it is enough to have this same number of partial histogram vectors to make this dependence disappear. To overcome the overhead from the synchronizations, we have chosen to use two new experimental features in the OmpSs@FPGA ecosystem: in-FPGA task creation [26] and in-FPGA dependence management [27]. As can be seen in Algorithm 6, not only are Aggregate and Distance tasks implemented in the FPGA, but also the FPGA_Main task that creates the other tasks. This prompts the OmpSs framework to include the Picos hardware runtime [28] in the FPGA which allows all the synchronization to be done locally inside the FPGA.

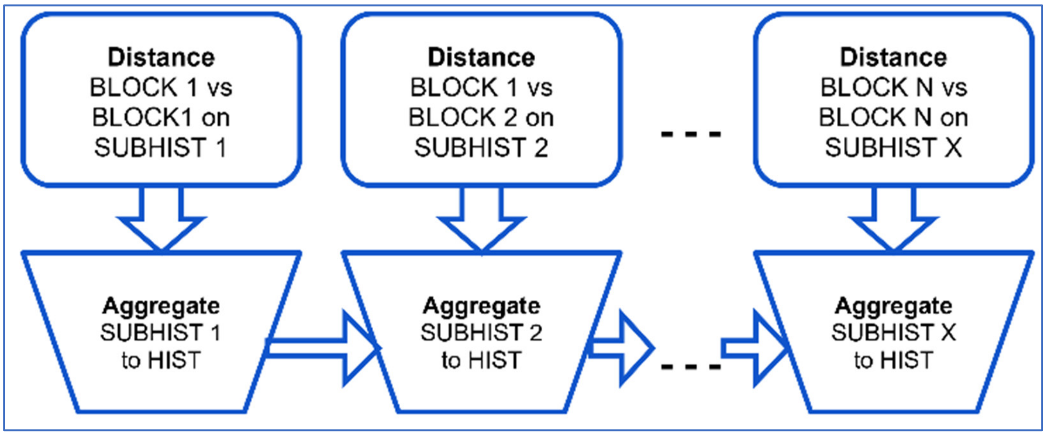

Figure S3. Task dependence pattern created by Algorithm 6.

Figure S4 shows the scheme of the design. As it can be seen, Nanos runtime executing in the symmetric multiprocessors (SMPs) copies the application data from the SMP memory to the FPGA memory (1) and sends the first FPGA_Main task to the FPGA (2). At this moment, the hardware runtime starts working and the Picos OmpSs Manager (POM) forwards the task to the corresponding hardware accelerator in the FPGA (3). The FPGA_Main kernel starts executing and creating tasks that return to the POM (4). The tasks have dependencies that ensure the correct execution order (as shown in Figure S3), so Picos Daviu (the current version of Picos: an evolution of the previous Picos++) receives the tasks (5) and, as soon as their data are ready, sends them to execution (6) in the corresponding accelerator (the Distance (7) or Aggregate (8) kernel). The hardware runtime ensures that only the tasks with data ready are executed, and, at the same time, distributes them among all the available hardware accelerators, profiting the application parallelism transparently for the programmer. When all the tasks have finished executing, the hardware runtime notifies the SMPs (9) and Nanos copies back the results (10).

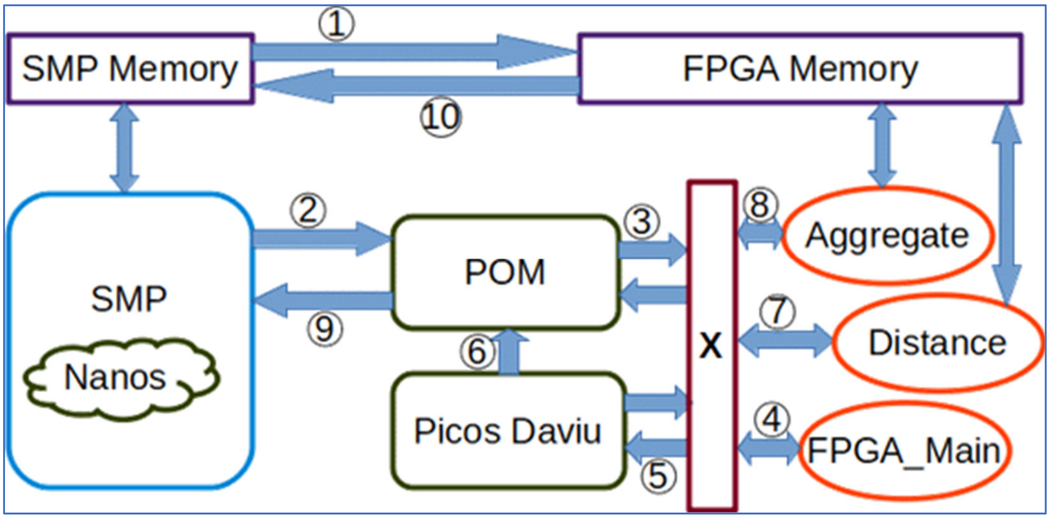

Figure S4. Application flow with Picos OmpSs manager.

1.2.1. Execution Trace

Figure S5 shows a trace [29] of the execution of a small 20,000-particle example problem using three Distance accelerators working at 250 MHz each. The trace details are measured with in-FPGA counters that register events in real time. The OmpSs@FPGA environment is able to add instrumentation hardware (cycle counters and communication infrastructure) automatically into any program, showing exactly how the system behaves. Figure S5 shows in each row the work done by one of the physical resources of the system during the execution of the program. The SMP0 to SMP3 are the four ARM cores of the system while FPGA_Main, Distance and Aggregate are the kernels implemented in hardware inside the FPGA fabric. There are physically three Distance kernels in the FPGA operating in parallel. The computation is started by SMP2 (the task indicated by the light blue bar), which sends the data to the FPGA. After that, the FPGA_Main kernel takes care of creating the different Distance and Aggregate tasks; these are executed in parallel if there are enough resources available. Each small green flag represents the beginning or end of one task. The different Distance kernels continuously operate on the data all through the program time while the Aggregate kernel performs a fast sequence of small tasks (three, one for each Distance task) after each Distance task finishes.

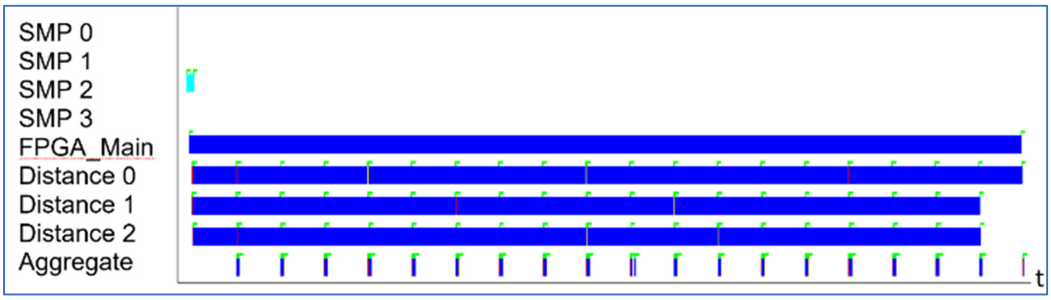

Figure S5. Trace execution of 20,000-particle pp-distance problem using three accelerators with 36 unroll factors. Flags represent beginnings or ends of tasks. Light blue bar shows initiation of computation by symmetric multiprocessor (SMP) 2.

Figure S5 also shows two interesting effects. The first is that there can be some load unbalance in the algorithm. The last Distance task is performed by the Distance0 kernel when all other tasks have already finished. After that, a final aggregate task is also performed before finishing the FPGA_Main task and the computation. Also, the SMPs are empty (waiting) while the FPGA computes the whole algorithm. We have tested the possibility of doing the Aggregate task, or even some Distance tasks, on the kernels. However, although this approach is automatically implemented by the OmpSs@FPGA environment, the data movement and performance difference between the cores and FPGA kernels makes this approach more time-consuming than doing everything in the FPGA.

Figure S6 is a more detailed version of Figure S5 where the copying time can be observed. The blue color represents the time where the kernels are computing, while the red color represents the copying of data into the accelerators and the yellow color represents the copying of data out of the accelerators. Two effects can be observed: first, that the data copies only use a small amount of the application time; second, that the hardware runtime is saving some copies when the data are already in the accelerators. As can be seen, the red bars in the tasks that start at the end of the trace in Figure S6 have different lengths than those at the beginning of the trace: when one of the two blocks has already been used in the previous Distance computation, it is not copied again. The same effect can be seen in the input and output copies of the middle Aggregate task of each of the three consecutive ones shown.

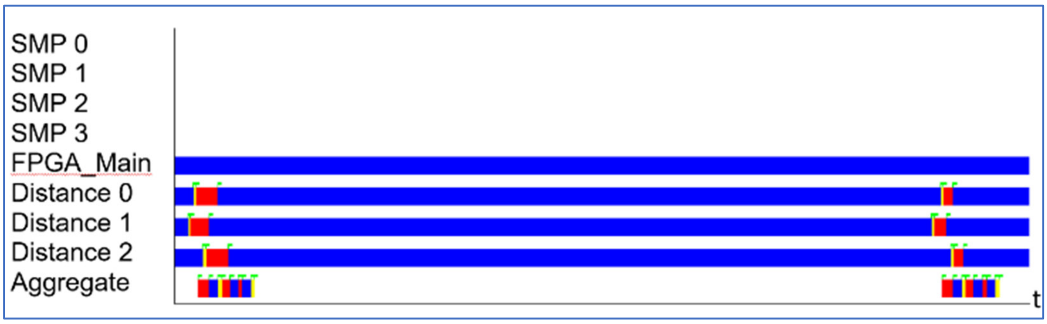

Figure S6. Trace execution of 20,000-particle example pp-distance problem using three accelerators with 36 unroll factors. Blue: kernel computation. Red: copying into accelerators. Yellow: copying out of accelerators. Flags represent beginnings or ends of tasks.

2. Optimized OpenCL Implementation

The OpenCL kernel implementation discussed in section 3.1. of the article has been analyzed and a set of code modifications has been implemented to remove the performance bottlenecks identified above.

First, the number of accesses to global memory has been greatly reduced by preloading data tiles to local memory before using them for computing. OpenCL local memory is

implemented in on-chip RAM blocks in the FPGA and has much higher throughput and much lower latency than global memory implemented in the external DDR. Besides, local memory can be shaped to allow multiple simultaneous read/write accesses to data and can be accessed randomly with no performance penalty. Tiles are preloaded through high efficiency sequential reads from global memory.

In the original implementation of the pp-distance algorithm, each datum is indexed in the data vector by the number of times it is read from global memory. Hence, the total number of required data reads is equal to N(N+1)/2. For two million data points, this is approximately two trillion data-point reads, corresponding to 32 TB of data for single-precision floating-point values. If tiles of size T are preloaded to local memory, the number of reads from global memory is reduced by a factor equal to T. With T=256, the amount of read data drops to 128 GB, solving the memory bandwidth problem reported earlier.

A second optimization has been introduced: coalescing the four components of a data point – the three spatial coordinates and the electronic weight – into a single record of type float4. That allows the kernel to perform a single wider read instead of four reads from competing locations, reducing the number of accesses and further improving the pattern of access to the global memory system.

A different problem limiting the performance of the original kernel implementation is throughput limitation caused by loop-carried dependency. The OpenCL compiler aims to implement a kernel as a deeply pipelined data path, one that should ideally be able to start processing a new dataset in the innermost algorithm loops on every clock cycle. However, loop-carried dependencies can force the compiler to increase the initiation interval, which is the number of clock cycles which must pass before a new dataset can enter a loop.

In the pp-distance algorithm, the aggregation step introduces a loop-carried dependency. A new iteration of the operation

accumulator[distancei,j] += weighti,j

cannot start before the previous one is completed, because the result of the previous iteration might be used by the next. As a result, the kernel can process one data pair every L clock cycles, where L is the total latency of the accumulator load-add-store operation sequence. The value of L depends on the target clock frequency set for the kernel at compile time; a higher frequency corresponds to a higher latency, since the compiler adds more pipeline registers to the processing chains to meet the frequency target.

This loop-carried dependency can be removed by creating L copies of the accumulation memory, looping accumulation operations on the L copies, and summing up results at the end. When this is done, accumulations on each memory copy occur every L clock cycles and a new accumulation can take place at every clock cycle, leading to an efficient use of the data-path logic while maximizing the data-path throughput.

Some reflection is now needed on the trade-off between throughput and resource usage. Both loop-carried dependency removal and spatial unrolling have a cost in terms of internal RAM; unrolling UF times requires an accumulation memory UF times larger, while removing a loop-carried dependency from a feedback path with latency L requires an accumulation memory L times larger. If both optimizations are applied, the accumulation memory size increases by a factor equal to UF*L. On the other hand, the kernel throughput is a function of three variables – the UF, initiation interval of the innermost loop (equal to L in the original kernel and to 1 in the optimized kernel), and kernel clock frequency F. The throughput of the original kernel implementation is bounded by UForig*Forig/Lorig, while for the optimized kernel implementation it is bounded by UFopt*Fopt. The speed-up factor coming from optimization is therefore equal to (UFopt/UForig)*(Fopt/Forig)*Lorig.

Note that the accumulation latency of the optimized kernel Lopt does not appear in the throughput speed-up equation. However, it does appear in the implementation-cost

equation, since the amount of internal RAM required by the optimized kernel is proportional to UFopt*Lopt. Reducing Lopt makes more internal RAM available for increasing UFopt, which has a positive impact on the throughput. Lopt can be reduced by lowering the target Fopt; using this trade-off allows doubling the UF with a very limited reduction of the actual kernel frequency, leading to a significant net gain in performance.

Starting from the above considerations, a further improvement in the kernel performance has been achieved with a change in the kernel architecture. In the original implementation, weight accumulation is performed in double-precision floating point, and the minimum possible latency of the accumulation loop is the latency of the double-precision floating-point adder in that loop. In the modified implementation, partial weight accumulations are performed using 40-bit integer arithmetic; results are periodically accumulated using double-precision floating-point arithmetic. Since the latency of a 40-bit integer adder is much lower than the latency of a double-precision floating-point adder, Lopt is significantly reduced and a higher UF can be achieved. Experiments show that this approach introduces no difference in the computed results for the two-million-point case.

The optimized kernel (algorithm 7) has been tested on two FPGA platforms – the Intel Programmable Acceleration Card with Intel Arria 10 GX FPGA and the Intel FPGA Programmable Acceleration Card D5005, featuring an Intel Stratix 10 SX FPGA [12]. The kernel for the FPGA is compiled with the Intel FPGA SDK for OpenCL version 20.1 using as backend the Intel Quartus compiler version 17.1.1 for the first card and version 18.1.2 for the second.

Synthesis experiments have been run for different target frequencies and UFs to determine the best fit on each platform. Implementation details of the configurations used for these tests are shown in Table S1. The results are shown in Table S2 and in Figure S7, Figure S8 and Figure S9. In Table S1, configurations 4 and 7 use 40-bit integer partial accumulation, while all others use full double precision floating point accumulation.

The resources used, that appeared in Table S1 are: Adaptative Look-Up-Table (ALUT); Multiply, Multiply-add, and Multiply-accumulate functions (DSP); Registers (REG); and Block RAMs of 20Kb each (M20K).

Table S1. Implementation details of the tested configurations.

	PLATFORM	UF	L	TARGET FREQUENCY	ACTUAL FREQUENCY	FPGA RESOURCE USAGE			
				[MHz]	[MHz]	ALUT	REG	DSP	M20K
1	Arria 10 PAC	1	13	default	294	117,524	134,709	11	455
2	Arria 10 PAC	8	16	default	251	163,242	172,443	81	1516
3	Arria 10 PAC	16	14	200	232	223,681	218,283	161	2354
4	Arria 10 PAC	32	5	default	244	254,034	285,579	320	1572
5	D5005 PAC	32	21	270	280	282,926	549,841	305	7700
6	D5005 PAC	64	16	180	241	436,679	715,900	609	11,146
7	D5005 PAC	128	8	270	255	724,479	1,285,009	1216	7479

Table S2. Optimized kernel time in seconds for the different executions.

NUMBER OF PARTICLES	CONFIGURATION						
	1	2	3	4	5	6	7
**20,000**	0.708	0.106	0.059	0.035	0.030	0.020	0.017
**200,000**	68.409	10.021	5.424	2.803	2.266	1.321	0.882
**2,000,000**	6820.520	998.843	540.457	274.020	223.554	130.012	81.573

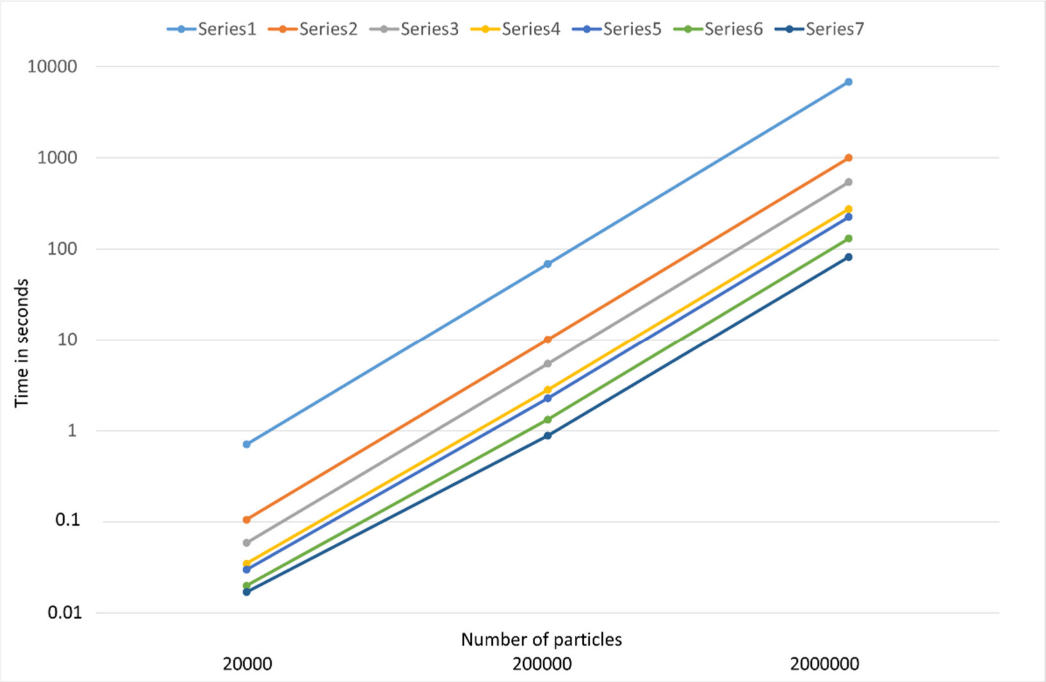

Figure S7. Execution time for the optimized kernel configurations as a function of the number of particles.

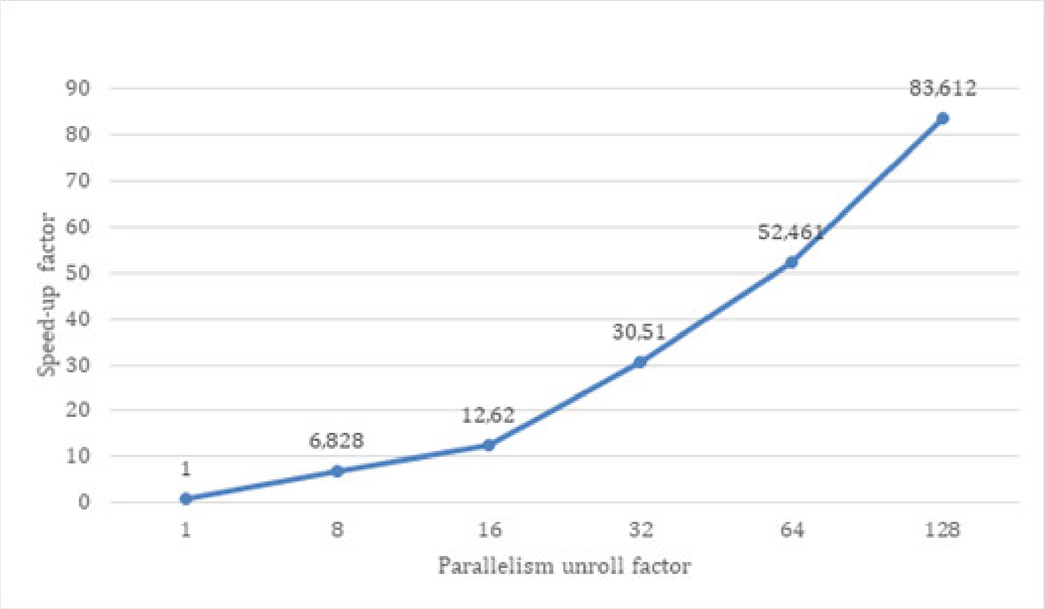

Figure S8. Speed-up factor for different unroll factors, optimized kernel.

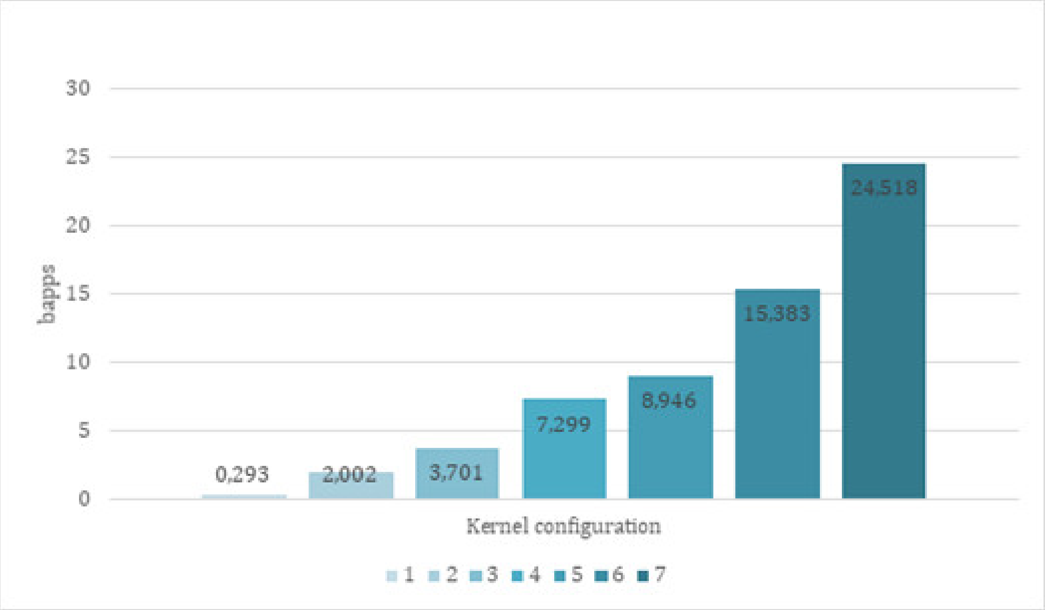

Figure S9. Performance, in bapps, of different optimized kernel configurations.

Algorithm 1 OmpSs@FPGA code with experimental features

```
#pragma omp target device(fpga) num_instances(NUMACC)
#pragma omp task in([NB]h_B1A, [NB]h_B2A, [NB]h_B3A, [NB]h_B4A)  \
    in([NB]h_B1B, [NB]h_B2B, [NB]h_B3B, [NB]h_B4B) out([F]subhistogram)
void Distance (float  *h_B1A, float  *h_B2A, float  *h_B3A, float  *h_B4A,
        float  *h_B1B, float  *h_B2B, float  *h_B3B, float  *h_B4B, int *subhistogram) {
  initialize UNROLL partial_histograms to 0
  for (int j = 0; j < SIZE_NB; j++) {
     for (int i = 0; i < SIZE_NB; i+=UNROLL) {
        compute UNROLL distances j,i between particles j and i to i+UNROLL-1
        compute UNROLL electronic weights j,i between particles j and i to i+UNROLL-1
        aggregate the value of UNROLL weights j,i to UNROLL partial_histograms in position distance
j,i
     }
  }
  aggregate UNROLL partial_histograms to subhistogram
}

#pragma omp target device(fpga) num_instances(1)
#pragma omp task in([F]subhistogram) inout([F]histogram)
void Aggregate(int *subhistogram, double *histogram) {
    aggregate subhistogram to histogram
}

#pragma omp target device(fpga) \
     copy_in([num_particles]h_B1, [num_particles]h_B2, [num_particles]h_B3) \
     copy_in([num_particles]h_B4) copy_inout([NUMACC*F]subhistogram, [F]histogram)
#pragma omp task
void FPGA_Main (int num_particles, int NUMACC, float *h_B1, float *h_B2,
        float *h_B3, float *h_B4, int *subhistogram, double *histogram) {
     int hist_index = 0;
     for (int nbi=0; nbi<num_particles; nbi=nbi+SIZE_NB) {
        for (int nbj=nbi; nbj<num_particles; nbj=nbj+SIZE_NB) {
               Distance(h_B1 + nbi, h_B2 + nbi, h_B3 + nbi, h_B4 + nbi, h_B1 + nbj, h_B2 + nbj,
h_B3 + nbj, h_B4 + nbj, subhistogram + hist_index*F, nbi==nbj);
               Aggregate(subhistogram + hist_index*F, histogram);
               hist_index = (hist_index+1)%NUMACC;
        }
     }
     #pragma omp taskwait
}
```

```
int main (...)  {
   ...
   FPGA_Main(num_particles, NUMACC, h_B1, h_B2, h_B3, h_B4, subhistogram, histogram)
   #pragma omp taskwait
}
```

Algorithm 2 Program to generate distribution of pseudo-particles (Visual Basic)

```
Rem generates points inside a sphere with a shell of different density
Dim posi(3, 2600000), x(10), randa(3)
Dim vec(2, 120000)
Dim sortida$(10)
pi = 3.141592
Rem initialize the particle count counter
part = 1

'external radius of the sphere
re = 10.8: re2 = re * re
'internal radius
ri = 5.4: ri2 = ri * ri
'electronic density of the shell and the interior
de = 0.07
di = -0.08
partfi = 2000000
partf = partfi
For i = 1 To partf
Loop1:
Call aleat(randa)
For j = 0 To 2
posi(j, i) = (randa(j + 1) - 0.5) * (re * 2)
Next j
posi(j, i) = de
r2 = (posi(0, i) ^ 2 + posi(1, i) ^ 2 + posi(2, i) ^ 2)
If r2 > re2 Then GoTo Loop1
If r2 < ri2 Then
k = k + 1
posi(j, i) = di
End If
Next i
b$ = "FilePath\FileName" + ".dat"
Open b$ For Output As #1
For i = 1 To partf
For j = 0 To 2
```

```
If posi(j, i) < 0 Then
sortida$(j) = Format(posi(j, i) / 1000000000#, ".000000E+00")  'these units must be in accordance with those used in Fourier Transform,.
Else
sortida$(j) = "+" + Format(posi(j, i) / 1000000000#, ".000000E+00")
End If
Next j
If posi(j, i) < 0 Then
sortida$(j) = Format(posi(j, i), ".000000E+00")
Else
sortida$(j) = "+" + Format(posi(j, i), ".000000E+00")
End If
sortida$(5) = sortida$(0) + " " + sortida$(1) + " " + sortida$(2) + " " + sortida$(3) + " "
Print #1, sortida$(5)
Next i
Close (1)
end

Sub aleat(randa)
Static js, jk, jr
'Generates triplets of uniform pseudorandom numbers from 0 to 1
    js = (js * 3877 + 29573) - 139968 * Int((js * 3877 + 29573) / 139968)
    jk = (jk * 3613 + 45289) - 214326 * Int((jk * 3613 + 45289) / 214326)
    jr = (jr * 1366 + 150889) - 714025 * Int((jr * 1366 + 150889) / 714025)
    randa(1) = 0.000007144902 * (js)
    randa(2) = 0.0000046657895 * (jk)
    randa(3) = 0.0000014005112 * (jr)
End Sub
```

Algorithm 3 PP-distance direct implementation

```
// LOOP 1
for j = 0, N do
  // LOOP 2
  for i = j, N do
    compute distance j,i between particles j and i
    compute electronic weight j,i between particles j and i
    aggregate the value of weight j,i to histogram in position distance j,i
  end for
end for
```

Algorithm 4 Kernel implementation of algorithm 3 with unroll factor UF

```
// LOOP 1
```

```
for j = 0, N do
  // LOOP 2
  threshold = int (( N − j ) / UF ) * UF
  for l = j, threshold, UF++ do
   # pragma unroll UF
    for m = 0, UF do
      k = l + m
      compute distance i,k between particles j and k
      compute electronic weight j,k between particles j and k
      aggregate the value of weight j,k to histogram in position distance j,k , to vector m
    end for
  end for
  // remaining cases
  for k = j + threshold, N do
    compute distance j,k between particles j and k
    compute electronic weight j,k between particles j and k
    aggregate the value of weight j,k to histogram in position distance j,k , to first vector (any
of the UF vectors)
  end for
end for
// final aggregation loop
for f = 0, F do // F is the number of frequency vector elements
  for m = 1 to UF do
    aggregate the value of histogram f from vector m to the vector to be returned to CPU
  end for
end for
```

Algorithm 5 OmpSs target directive and kernel declaration at C main program

```
// ❶
#pragma omp task device(opencl) ndrange(1, 1, 1) copy_deps in(h_B1[0;N]) \
                              in(h_B2[0;N]) \
                              in(h_B3[0;N]) \
                              in(h_B4[0;N]) \
                              out(h_A[0;F])
// ❷
__kernel void My_kernel (
        int N,
        int F,
        __global float  * restrict h_B1,
        __global float  * restrict h_B2,
        __global float  * restrict h_B3,
        __global float  * restrict h_B4,
```

```
        __global double * restrict h_A);
```

Algorithm 6 OmpSs@FPGA task example

```
#pragma omp target device(fpga) num_instances(NUMACC)
#pragma omp task in([NB]h_B1A, [NB]h_B2A, [NB]h_B3A, [NB]h_B4A)  \
    in([NB]h_B1B, [NB]h_B2B, [NB]h_B3B, [NB]h_B4B) inout([F]histogram)
void Distance (float  *h_B1A, float  *h_B2A, float  *h_B3A, float  *h_B4A,
        float  *h_B1B, float  *h_B2B, float  *h_B3B, float  *h_B4B, double *histogram) {
  initialize UNROLL partial_histograms to 0
  for (int j = 0; j < SIZE_NB; j++) {
     for (int i = 0; i < SIZE_NB; i+=UNROLL) {
        compute UNROLL distances j,i between particles j and i to i+UNROLL-1
        compute UNROLL electronic weights j,i between particles j and i to i+UNROLL-1
        aggregate the value of UNROLL weights j,i to UNROLL partial_histograms in position distance
j,i
     }
  }
  aggregate UNROLL partial_histograms to histogram
}

int main (...)  {
  int fidx = 0;
  for (int nbi=0; nbi<num_particles; nbi=nbi+SIZE_NB) {
    for (int nbj=nbi; nbj<num_particles; nbj=nbj+SIZE_NB) {
      Distance (h_B1 + nbi, h_B2 + nbi, h_B3 + nbi, h_B4 + nbi, \
          h_B1 + nbj, h_B2 + nbj, h_B3 + nbj, h_B4 + nbj, histogram + fidx*F);
      fidx = (fidx+1)%NUMACC;
    }
  }
  #pragma omp taskwait
  aggregate NUMACC histograms to final_histogram
}
```

Algorithm 7 PP-distance optimized kernel implementation

```
lat = 0;
ntiles = N / TILE_SIZE;
if (N % TILE_SIZE) ntiles++;
// LOOP 1
for t1 = 0, ntiles do
  load first data tile in local memory
  // LOOP 2
  for t2 = t1, ntiles do
```

```
    load second data tile in local memory
    // LOOP 3
    for i = 0, TILE_SIZE do
      // LOOP 4
      for k = 0, TILE_SIZE/UNROLL_FACTOR do
#pragma unroll UNROLL_FACTOR
        for l = 0, UNROLL_FACTOR do
          j = k*UNROLL_FACTOR+l;
          compute distance j,i between particles j and i from local memory
          compute electronic weight j,i between particles j and i from local memory
          if (((t1 != t2) || (i <= j)) && ((t1*TILE_SIZE+i) < N) && ((t2*TILE_SIZE+j) < N))
            aggregate the value of weight j,i to subhistogram[l,lat] in position distance j,i
          end if
        end for
        lat = (lat == L-1) ? 0 : lat+1;
      end for
    end for
  end for
end for
for i = 0, F do
  aggregate position i from all subhistograms to the vector to be returned to CPU
end for
```
